# Wheat-yellow pumpkin composite flour: Physico-functional, rheological, antioxidant potential and quality properties of pan and flat bread

**DOI:** 10.1016/j.sjbs.2022.02.040

**Published:** 2022-02-26

**Authors:** Amani H. Aljahani

**Affiliations:** Department of Physical Sport Science, College of Education, Princess Nourah bint Abdulrahman University, P.O. Box 84428, Riyadh 11671, Saudi Arabia

**Keywords:** Yellow pumpkin, Rheological, Antioxidant, Organoleptic, Bread, YP, Pumpkin powder, RVA, Rapid Visco-Analyser, AACC, American Association of Cereal Chemists, OHC, Oil holding capacity, WHC, Water holding capacity

## Abstract

The impact of yellow pumpkin powder (YP) substitution (5, 10, and 15%) on wheat flour's physico-functional, pasting, gel texture, and dough rheology was studied. Moreover, the nutritional, organoleptic properties and bioactivity of the composite pan and pita bread were evaluated. An improved water holding capacity was noticed for the blended flour than for control. The pasting parameters were declined significantly (p < 0.05) with increasing YP. Composite flours presented a softer gel texture in the presence of YP. Reduced water absorption, increased dough development time, and lower stability for combined flour dough. The bread with YP depicted increased protein, fat, fiber, and mineral contents, while a reduced volume and specific volume were noticed for pan bread. YP incorporated 5% and did not compromise pan and pita bread's color and overall acceptability. Additionally, composite bread depicted higher total phenolics with enhanced antioxidant activities at the higher substitution of YP.

## 1 Introduction

Wheat being a staple in many parts of the globe, the production and consumption of wheat bread are major sources to quench the population's energy needs. Wheat bread is relished for its tempting organoleptic parameters, cost competitiveness, and ready-to-eat characteristics. Additionally, it is an evident source of macro and micronutrients, such as carbohydrates, proteins, fiber, vitamins, and minerals (Millar et al., 2019). However, the growing public awareness and epidemiological evidence towards diet-related ailments urge the baking industry to develop nutritionally rich and closer to nature with sufficient storage stability (Silva et al., 2011). Hence, to cope with the consumer demands of so-called super foods rich in proteins, fibers, or bioactive agents, bakery products are supplemented with non-cereal ingredients from various fruits, vegetables, and beans. Generally, fruits and vegetable products such as peels, purees, and seeds are abundant in nutritional and non-nutritional but functional components that could augment the physico-functional and nutritional profile of the bread upon addition. In developing countries, wheat flour substitution to make composite flour could also reduce wheat import to a certain extent while maximizing the utility of other local crops (See et al., 2007).

*Cucurbita moschata* is one of the cultivars under the pumpkin family called *Cucurbitaceae*. Among cucurbitaceous vegetables, pumpkin has been appreciated for its high yield, long storage life, and high nutritive value (Dhiman et al., 2009). Pumpkin is extensively grown in tropical and subtropical countries and consumed as steamed, boiled, or processed to food items such as soup. It is considered a valuable source of β-carotene (Bhaskarachary et al., 2008), polyphenols, flavonoids, minerals, and vitamins, e.g., B6, K, thiamine, and riboflavin (Terazawaet al., 2001, Nawirskaet al., 2009). The richness of bioactive such as carotene, phenolics, and flavonoids in the pumpkin instigate its use in various food products. The dried pumpkin powder is a processed product that could be easily stored for longer and employed in manufacturing formulated foods. This flour can be used to supplement cereal flour in bakery products, soups, dairy products, and instant noodles to improve the nutritional, physical, and orosensory qualities of the properties of the food (Seeet al., 2007, Yoket al., 2016). Pongjanta et al. (2006) added pumpkin powder for developing chiffon and butter cakes, cookies, and sweet bread. Batista et al. (2018) produced cupcakes from wheat-pumpkin seed composite flour stuffed with carob beans for enhanced protein content.

The substitution with pumpkin-seed flour up to 50% produced cake similar to the wheat flour with good texture and cohesion. Similarly, pumpkin puree was incorporated in the ice cream for phenolic enrichment. The phenolic-rich ice cream's rheological and sensory properties (color and flavor) were significantly improved, and a greater antioxidant activity was observed (Mehditabar et al., 2019). Yildiz and Dzcan (2019) manufactured pumpkin-enriched yogurt with improved texture and antioxidant properties. Moreover, relatively higher ascorbic acid, total phenolics, and carotenoids were observed due to the substitution of the pumpkin fibers. It has been employed in cake mixes with enhanced flavor and color appeal (Salehi and Aghajanzadeh, 2020). Thus, based on these studies, it could be suggested that pumpkin powder may enrich pan and pita bread to enhance the nutritional, functional, and sensory properties. Thus, the objective of the current study was to scale the effect of the yellow pumpkin powder (YP) on the wheat flour dough rheology, pasting, and gel textural properties. Moreover, the impact on the bread physico-functional and organoleptic properties was also evaluated.

## 2 Materials and methods

### 2.1 Raw materials

Yellow pumpkin fruits *Cucurbita moschata*, wheat flour, yeast, salt, sugar, shortening, and improver were bought from the local supermarket, Riyadh, Saudi Arabia.

### 2.2 Preparation of yellow pumpkin powder

The cleaned yellow pumpkin fruits were cut, seeds were removed. Seedless fruits wear cut into 2-mm-thick slices and freeze-dried. The dried pumpkin was milled, sieved through a 60-mesh sieve, and stored in a refrigerator for further use.

### 2.3 Preparation of flour blends

To prepare Wheat flour and YP composites, the wheat flour was replaced with YP at 5%, 10%, and 15% w/w basis.

### 2.4 Water and oil holding capacities of flour blends

Water and oil holding capacities (WHC/OHC) were measured according to Traynham et al. (2007) with some modifications. The samples were placed in a centrifuge tube and suspended in 28.5 mL of water (WHC) or 28.5 mL of oil (OHC). Samples were shaken, rested, centrifuged, then decant the supernatant. The WHC and OHC were calculated as follows:

WHC/WOC (g/g) = (W1 − W0)/W0, where W0 is the initial weight (g) of the samples and W1 is the final weight (g) of samples.

### 2.5 Pasting properties of flour blends

A Rapid Visco-Analyser (RVA) (Newport Scientific, Sydney, Australia) was used to study the pasting behavior of wheat flour-YP blends. The flour samples (3.5 g at 14% moisture basis) were weighed directly in RVA aluminum pans, and distilled water was added to 28 g as total weight. The suspension was loaded in RVA and stirred. Then speed was decreased. The obtained slurry was equilibrated after that heated; it was then held. The suspension was cooled and then grabbed. Thermocline® for Windows (Newport Scientific, Sydney, Australia) was used for processing viscosity data (Mahmood et al., 2018).

### 2.6 Gel texture analysis

The gel obtained from the RVA experiment was stored overnight at room temperature in a 25-mL beaker with a 35-mm height and 30-mm internal diameter. The gel was compressed using a Brookfield CT3 Texture Analyzer (Brookfield Engineering Laboratories, Inc., Middleboro, MA, USA) in two penetration cycles at a speed of 0.5 mm/s over a distance of 10 mm using a 12.7-mm-wide and 35-mm-long cylindrical probe. Gel texture parameters such as hardness, springiness, cohesiveness, and adhesiveness were recorded. According to the method given by Hussain et al. (2013), the gumminess was calculated as the product of hardness and cohesiveness.

### 2.7 Micro-doughLab study

Micro-doughLab 2800 (Perten Instruments, Sydney, Australia) was used to determine the optimum water absorption of flour blends to reach a peak of 500 BU using 4.00 ± 0.01 g samples with a 14% moisture basis. The masses of the sample and water were corrected based on the moisture contents of the samples. The manufacturer's rapid mixing protocol was used: the composite flour was mixed at 30 °C for 10 min at a constant speed of 64 RPM. A mixing curve was obtained that reflected the dough's bending properties: the peak (FU), water absorption (WA %), development time (min), stability (min), softening (FU), and mixing tolerance index (MTI; FU) (Hussain et al., 2013).

### 2.8 Pan bread preparation

The pan bread was prepared using the Approved Methods of the American Association of Cereal Chemists International (AACC, 2000) Method 10-10A. In brief, the ingredients Table 1. were mixed based on 700 g wheat flour (14% moisture content), 3% instant yeast, 3% shortening, 2% salt, 5% sugar, and 0.01% improver. YP (5%, 10%, and 15% w/w of wheat flour). The water was added based on the optimum flour absorption as determined by micro-doughLab. All ingredients were mixed using (Tyrone, model TR 202, UK) to optimize dough development time. The dough was formatted it was kept in a fermentation cabinet for 30 min. Then dough balls were sheeted, divided, and fermented again for 20 min. Finally, the dough balls were molded and shifted to the baking pans for the final proofing for 30 min under the same conditions. After fermentation, the loaves were baked for 20 min at 225 °C using the rotary oven (National Co. Ltd., Kyoto, Japan).

**Table 1:** Pan and pita bread ingredients.

Ingredients	Pan bread				Pita bread			
	Control	5%	10%	15%	Control	5%	10%	15%
Wheat flour (g)	700	665	630	595	700	665	630	595
YP* (g)	–	35	70	105	–	35	70	105
Water (mL)	434	430.5	433	388.2	434	430.5	433	388.2
Yeast (g)	7	7	7	7	7	7	7	7
Sugar (g)	35	35	35	35	35	35	35	35
Salt (g)	7	7	7	7	7	7	7	7
Shortening (g)	21	21	21	21	–	–	–	–
Improver (g)	0.07	0.07	0.07	0.07	0.07	0.07	0.07	0.07

* YP = yellow pumpkin

### 2.9 Pita bread preparation

The pita bread was made according to Mousa and Al-Mohazea, (1987). In summary, the ingredients were mixed based on 700 g wheat flour (14% moisture content) with (3, 5, 1, 0.01%) instant yeast, sugar, salt, and improver, respectively. YP (5%, 10%, and 15% w/w of wheat flour). The water was added, and the ingredients were mixed to form the dough. The fermented dough was formed into balls by hand and fermented again. Then the dough was flattened, thickness, and fermented. The bread was baked at 350 °C for 2 min.

### 2.10 Quality measurements

The baking bread samples were cooled to room temperature for 1 h and weighed. The volume of bread samples was determined by the rapeseed displacement method. Specific volume (cm^3^/g) was calculated according to Koca and Anil (2007). An Aqua Lab water activity meter (CX-2, Decagon Devices Inc., Pullman, WA, USA) was used to determine the bread samples' water activity (wa).

### 2.11 Color measurements

Bread crust and crumb color were estimated in terms of ‘CIE L*a*b*’ parameters at room temperature by Hunter lab calorimeter (LabScan XE, USA). The L* indicates lightness, a* indicates redness, and b* indicates yellowness.

### 2.12 Proximate analysis

The moisture content, protein, fat, and ash for bread samples were conducted using Official Methods of Analysis (AOAC, 2006). The carbohydrate content was determined by calculation using the different methods:

%Total Carbohydrate = [100 − % (Protein + Fat + Moisture + Ash + Fiber)].

### 2.13 Total phenols, antioxidants, and radical scavenging activity determination

*Sample extraction:* one gram of bread samples was mixed with 25 mL of ethanol in a shaker. The blend was centrifuged وcollected and filtered. According to Wu et al. (2007), total polyphenols were determined with adjustment. Gouveia and Castilho (2011) mothed was used to determine of the *ferric reducing antioxidant power (FRAP****)*** and *radical scavenging activity DPPH (2,2-diphenyl-1-picrylhydrazyl)* was performed according to Akillioglu and Karakaya (2010). According to Gouveia and Castilho (2011), *radical scavenging activity ABTS* 2,2′-azino-bis (3 ethylbenzothiazoline-6-sulphonic acids was determined.

### 2.14 Sensory evaluation

Sensory analysis of bread samples was performed by 20 semi-trained panelists. The sensory attributes of the pan and pita bread were assessed using a 9-point hedonic scale (9 = like extremely, 1 = dislike extremely. Panelists were asked to determine crust color, symmetry, broken shard, crumb color, grain, texture, flavor, and overall acceptability of pan bread whereas crust color, uniformity, break and shred, crumb color; internal appearance: tactile crumb texture, flavor, overall acceptability attributes of pita bread.

### 2.15 Statistical analysis

Triplicate was done for all measurements. One-way analysis of variance and Duncan's multiple range test (at p ≤ 0.05) was used with the PASW® 18 software to compare the mean values.

## 3 Results

### 3.1 Water and oil holding capacities of flour blends

**Fig. 1:**
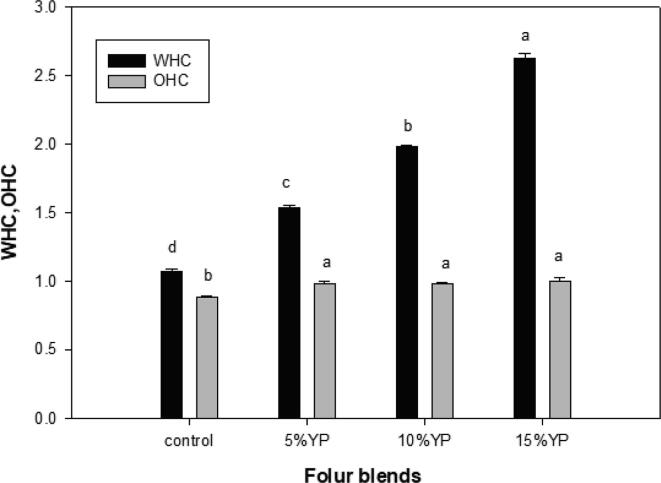
Water and oil holding capacities of flour blends.

The WHC of powdered material is given by the amount of water that can be retained under centrifugal or compressive forces. Fig. 1 represents the composite flours' WHC and oil holding capacity (OHC). Substituting YP significantly increased the WHC with an almost twofold increase for 10% YP substitution. Increasing the YP substitution from 5% to 15% also increased the WHC. A high WHC is an indirect indicator of the thickening ability of the flour.

### 3.2 Pasting properties of flour blends

The pasting properties of the composite flours are presented in Table 2. The pasting properties are used to estimate the flour performance during processing and possible staling of the baked bread (Collar 2003). The peak viscosity remained at 2281–2824 cp for the composite flours. Adding YP significantly reduced the peak viscosity (p < 0.05); in particular, 15% YP reduced the viscosity by 19%.

**Table 2:** Effect of yellow pumpkin (YP) substitution on the pasting properties of wheat flour.

**Sample**	**Peak viscosity(cP)**	**Breakdown (cP)**	**Final viscosity (cP)**	**Setback (cP)**	**Pasting temperature(^0^C)**
**Control**	2824.66 ± 38.73a	1157.33 ± 3.7c	3607.33 ± 10.96a	1945.33 ± 46.18a	86.56 ± 0.05a
**YP 5%**	2678.66 ± 3.21ab	1225.00 ± 4.00a	3448.00 ± 3.60ab	1989.33 ± 8.02a	84.00 ± 0.47b
**YP 10%**	2531.33 ± 1.52bc	1186.00 ± 1.00ab	3312.33 ± 7.09bc	1971.66 ± 7.63a	83.99 ± 0.02b
**YP 15%**	2281.00 ± 9.53d	1125.33 ± 4.04c	2940.66 ± 5.50c	1786.00 ± 4.00b	84.10 ± 0.80b

Values followed by different letters were significantly different at p < 0.05.

### 3.3 Gel texture analysis

The texture of the composite flour gel was estimated according to parameters such as hardness, cohesiveness, adhesiveness, springiness, and gumminess Table 3. Hardness is the force required to deform the gel, and it is expressed in grams (g). The hardness of the composite flour gels ranged from 30.6 to 40 g, where softer gels were obtained with increased YP substitution (10% and 15%).

**Table 3:** Gel texture properties of cooked flour gels.

**Sample**	**Hardness (g)**	**Cohesiveness**	**Springiness**	**Adhesiveness (mJ)**	**Gumminess**
**Control**	40.00 ± 1.00a	0.52 ± 0.01a	14.00 ± 1.00a	0.50 ± 0.01ab	20.80 ± 0.67a
YP* 5%	40.66 ± 0.57a	0.48 ± 0.02a	13.30 ± 0.20a	0.60 ± 0.01a	19.52 ± 0.06ab
**YP 10%**	31.00 ± 1.00b	0.52 ± 0.03a	8.80 ± 0.10b	0.20 ± 0.01c	16.35 ± 0.57bc
**YP 15%**	30.66 ± 0.57b	0.51 ± 0.01a	13.50 ± 0.30a	0.30 ± 0.01c	15.64 ± 0.90c

* YP = yellow pumpkin; Values followed by different letters were significantly different at p < 0.05.

### 3.4 Micro-doughLab study

Optimizing the rheological properties of the dough guarantees bread with a good volume, low density, and uniform grain structure. To reach the stage of optimum development, water was added to the optimal absorption level. The water absorption (%) for the corrected peak (500 FU) decreased with increasing YP substitution. The effect was significant (p < 0.05) with increasing YP substitution Table 4.

**Table 4:** Micro-doughLab data of the wheat flour and the blends.

**Sample**	**Peak (FU)**	WA* (%)	**Development time (min)**	**Stability (min)**	**Softening (FU)**	**MTI (FU)**
**Control**	519.80 ± 9.1a	61.50 ± 1.10a	1.40 ± 0.10c	2.20 ± 0.20c	65.00 ± 3.00d	50.00 ± 9.03b
YP^**^ 5%	529.76 ± 3.0a	61.86 ± 2.61a	6.80 ± 0.40cb	1.00 ± 0.20d	134.63 ± 3.50c	80.00 ± 6.16a
**YP 10%**	505.50 ± 5.0b	59.53 ± 0.80a	8.33 ± 0.57a	3.23 ± 0.152b	145.60 ± 5.15b	72.33 ± 7.89a
**YP 15%**	480.00 ± 5.0c	55.46 ± 0.75b	9.00 ± 1.00a	5.66 ± 0.55a	151.48 ± 3.52a	45.00 ± 4.91b

*WA: Water absorption; * YP = yellow pumpkin; Values followed by different letters were significantly different at p < 0.05.

### 3.5 Bread quality

The bread volume indicates a foamy crumb structure, which appeals to consumers. The gluten network mainly provides the bread volume, which holds the gas during the final baking, and starch generally acts as filler. The bread volume, weight, specific volume, and water activity data are presented in Table 5. The pan and pita bread weights significantly increased with the YP substitution from 5% to 15%. This may be attributed to the increased WHC of the composite flour. However, the presence of YP significantly reduced the volumes of the pan and pita bread (p < 0.05). In the case of the pan bread, adding YP reduced the volume by 13%–45% compared with the control bread. This volume reduction may be attributed to the dilution of the gluten network by substituting the fiber-rich YP.

**Table 5:** Effect of (YP) substitution on pan and pita bread quality characteristics.

**Sample**	**Loaf weight(g)**	**Loaf volume (cm3)**	**Specific volume(cm3/g)**	**Water activity**
**Pan bread**				
**Control**	489.00 ± 1.00d	2580.00 ± 15.20a	5.28 ± 0.17a	0.693 ± 0.01c
**YP 5%**	505.66 ± 2.02c	2239.66 ± 3.05b	4.43 ± 0.98b	0.702 ± 0.02b
**YP 10%**	513.00 ± 2.00b	1400.00 ± 5.00d	2.73 ± 0.01d	0.706 ± 0.03b
**YP 15%**	517.33 ± 4.02a	1570.00 ± 12.01c	3.03 ± 0.02c	0.726 ± 0.03a
**Pita bread**				
**Control**	131.00 ± 1.00c	291.00 ± 15.20a	2.28 ± 0.08a	0.653 ± 0.01b
**YP 5%**	135.66 ± 2.02b	283.66 ± 3.05b	2.43 ± 0.02b	0.747 ± 0.02a
**YP 10%**	137.00 ± 2.00b	279.00 ± 5.00b	2.23 ± 0.01a	0.751 ± 0.01a
**YP 15%**	140.33 ± 4.02a	273.00 ± 12.01c	1.93 ± 0.09c	0.756 ± 0.03a

Values followed by different letters were significantly different at p < 0.05.

### 3.6 Bread color

The colors of the crust layer and internal foamy crumb for both the pan and pita bread were measured as a function of the L, a, and b parameters Table 6. The composite pan bread had a significantly lighter crust color than the control.

**Table 6:** Effect of (YP) substitution on pan and pita bread color characteristics.

**Sample**	**Crust**			**Crumb**		
	L*	a*	b*	L*	a*	b*
**Pan bread**						
**Control**	69.11 ± 0.09d	14.47 ± 0.01a	29.31 ± 0.06a	69.86 ± 0.0d	2.72 ± 0.01a	36.90 ± 0.03a
**YP 5%**	73.1 ± 0.61a	−2.5 ± 0.01c	17 ± 0.91b	79.4 ± 0.63a	−0.07 ± 0.08d	14 ± 0.31c
**YP 10%**	72.6 ± 0.63b	2.3 ± 0.31b	18 ± 0.17c	76.8 ± 0.9b	−0.87 ± 0.05c	15 ± 0.01b
**YP 15%**	70.0 ± 0.61c	−3.0 ± 0.03c	19 ± 0.21c	74.6 ± 0.5c	−2.00 ± 0.1b	16 ± 0.03b
**Pita bread**						
**Control**	81.81 ± 0.01a	9.99 ± 0.01 d	11.49 ± 0.01d	51.39 ± 0.02d	2.11 ± 0.01a	28.44 ± 0.01c
**YP 5%**	79.09 ± 0.02b	12.78 ± 0.02c	23.54 ± 0.02c	60.76 ± 0.04c	1.40 ± 0.09b	31.60 ± 0.06b
**YP 10%**	75.50 ± 0.01c	13.83 ± 0.03b	25.29 ± 0.06b	63.19 ± 0.05b	1.20 ± 0.01b	32.87 ± 0.03b
**YP 15%**	72.06 ± 0.08d	14.50 ± 0.02a	27.06 ± 0.08a	65.68 ± 0.2a	2.50 ± 0.02a	34.97 ± 0.01a

Means with the same letter were not significantly different (p < 0.05); *L: lightness, a: redness, b: yellowness.

### 3.7 Proximate composition

Table 7 presents the proximate composition of the pan and pita bread. Increasing the YP substitution from 5% to 15% significantly improved the fat, protein, carbohydrate, and ash contents in the pan and pita bread compared to the controls. However, the YP substitution had a greater effect on the proximate composition of the pan bread than the pita bread.

**Table 7:** Proximate compositions of pan and pita bread with (YP*) substitution.

**Sample**	**Moisture (%)**	**Protein (%)**	**Fat (%)**	**Ash (%)**	**Carbohydrate (%)**
**Pan bread**					
**Control**	8.42 ± 0.09a	10.60 ± 0.11c	2.17 ± 0.10d	2.72 ± 0.10d	76.08 ± 0.40a
**YP 5%**	8.13 ± 0.10b	11.41 ± 0.12a	2.44 ± 0.09c	2.89 ± 0.11c	75.14 ± 0.40b
**YP 10%**	8.27 ± 0.21ab	11.30 ± 0.30a	2.79 ± 0.01b	3.21 ± 0.10b	74.42 ± 0.42c
**YP 15%**	8.04 ± 0.09b	11.15 ± 0.10a	3.12 ± 0.08a	3.48 ± 0.01a	74.21 ± 0.29c
**Pita bread**					
**Control**	8.25 ± 1.71a	10.84 ± 0.1a	0.18 ± 0.02b	1.68 ± 0.01d	79.05 ± 0.2a
**YP 5%**	9.51 ± 0.19a	10.8133 ± 0.09a	0.20 ± 0.01b	1.91 ± 0.07c	77.86 ± 0.12a
**YP 10%**	8.83 ± 0.15 a	10.52 ± 0.09b	0.24 ± 0.01a	2.12 ± 0.10b	78.27 ± 0.1a
**YP 15%**	7.69 ± 0.01a	10.33 ± 0.11b	0.27 ± 0.01a	2.41 ± 0.19a	79.29 ± 0.33a

* YP = yellow pumpkin; Values followed by different letters were significantly different at p < 0.05.

### 3.8 Total phenols, antioxidants, and radical scavenging activity

Pumpkin is rich in various bioactive fractions such as carotenoids, ascorbic acid, tocopherols, flavonols, and phenolic acids (Kulczyński and Gramza-Michałowska, 2019a, Kulczyński and Gramza-Michałowska, 2019b). In this study, YP was substituted for wheat flour to enhance the functional and nutritional properties of the bread. Table 8 presents the total phenolics, free radical scavenging activity, and iron-reducing ability. Adding 5%, YP reduced the total phenolic contents of the pan and pita bread. However, increasing the YP substitution improved the phenolic content compared with the control, although the change was non-significant.

**Table 8:** Antioxidants of pan and pita bread with (YP*) substitution.

**Sample**	**T. Phenols(mg GAE /g)**	**DPPH (%)**	ABTS*(g Trolox/g)	**FRAB(g Trolox/g)**
**Pan bread**				
**Control**	5.95± 0.24c	6.15± 0.36a	0.05±0.00a	0.05±0.00b
**YP 5%**	5.26±0.16c	5.83± 0.00b	0.06±0.00b	0.05±0.00b
**YP 10%**	5.21±0.48c	6.18± 0.24a	0.05±0.00a	0.08±0.0a
**YP 15%**	5.37±0.33c	6.40± 0.13a	0.04±0.00c	0.05±0.00b
**Pita bread**				
**Control**	5.01± 0.24c	5.85± 023b	0.06±0.00b	0.05±0.00b
**YP 5%**	5.21± 0.04c	6.25± 0.11a	0.07±0.00a	0.04±0.00c
**YP 10%**	5.27± 0.29b	5.95± 0.31b	0.05±0.00c	0.07±0.00a
**YP 15%**	5.30± 0.00a	6.95± 0.12a	0.02±0.00d	0.04±0.00c

* YP = yellow pumpkin; Values followed by different letters were significantly different at *p* < 0.05

### 3.9 Sensory analysis

Sensory analysis is crucial to establishing a correlation between consumer preferences and the microstructure of the composite bread. The bread's sensory attributes and nutritional composition are greatly dependent on the composition, methods used for dough development, proofing, baking, and other additives. Bread organoleptic properties are generally divided into two sections due to the bread crust and crumb attributes. Table 9 presents the sensory analysis results. The bread appearance reflects the extent of baking and helps estimate the raw material and formulation. The most important attributes to the consumer for the bread crust are color and symmetry.

**Table 9:** Sensory attributes analysis of pan and pita bread with (YP*) substitution.

**Pan bread**							
**Samples**	**Crust color**	**Symmetry**	**Break shard**	**Crumb color**	**Grainand texture**	**Flavor**	**Overall acceptability**
**Control**	8.20 ± 0.78a	8.63 ± 0.11a	8.43 ± 0.21a	8.15 ± 0.12a	8.80 ± 0.31a	8.16 ± 0.18a	8.59 ± 0.16a
**YP 5%**	7.60 ± 0.61a	7.45 ± 0.61b	7.60 ± 0.21b	7.50 ± 0.52b	7.03 ± 0.33a	7.33 ± 0.32b	7.13 ± 0.36b
**YP 10%**	5.30 ± 0.48b	5.51 ± 0.22c	5.36 ± 0.18c	6.41 ± 0.26c	6.63 ± 0.16b	6.20 ± 0.42c	6.27 ± 0.92c
**YP 15%**	5.00 ± 0.37b	5.98 ± 0.46c	5.53 ± 0.32c	5.53 ± 0.13d	5.50 ± 0.22c	5.70 ± 0.68d	5.57 ± 0.38d
**Pita bread**							
	**External appearance**			**Internal appearance**			
**Samples**	**Crust color**	**Uniformity**	**Break and shred**	**Crumb color**	**Tactile crumb texture**	**flavor**	**Overall acceptability**
**Control**	8.20 ± 0.78a	8.50 ± 0.52a	8.90 ± 0.33a	8.90 ± 0.73a	8.80 ± 0.42a	8.30 ± 0.18a	8.27 ± 0.86a
**YP 5%**	7.23 ± 0.51b	7.86 ± 0.48b	7.87 ± 0.61b	7.10 ± 0.31b	7.06 ± 0.31b	7.16 ± 0.66b	7.73 ± 0.48b
**YP 10%**	6.40 ± 0.51c	6.39 ± 0.93c	7.33 ± 0.63b	6.64 ± 0.09c	6.48 ± 0.93c	6.20 ± 0.12b	6.69 ± 0.51b
**YP 15%**	5.90 ± 0.31d	5.90 ± 0.31d	5.10 ± 0.31c	5.90 ± 0.31d	5.76 ± 0.48d	5.70 ± 0.68c	5.16 ± 0.31c

* YP = yellow pumpkin; Values followed by different letters were significantly different at p < 0.05.

## 4 Discussion

### 4.1 Water and oil holding capacities of flour blends

The presence of relatively high amounts of carbohydrates in the form of soluble and insoluble fibers in the YP may have increased the WHC of the composite flours. In general, soluble fibers have a higher WHC than insoluble fibers (Mahmood et al., 2018). The OHC is defined as the oil retention by particulate matter after incubation and centrifugation under controlled conditions. All composite flours had similar OHCs. In contrast, Mahmood et al. (2018) reported that composite flours had a higher OHC than the control wheat flour.

### 4.2 Pasting properties of flour blends

The viscosity reduction might be related to the lower total starch content, lower swelling rate, and higher proteins and fibers in the composite flour with YP substitution (Jukić et al., 2019). Flour with a higher peak viscosity generally has a greater breakdown viscosity. The breakdown viscosity is an indirect indicator of a sample's shear and heat stability during the cooking process. However, a relatively high peak viscosity is favorable for products that require high elasticity and strength (Adebowaleet al., 2005, Ikegwuet al., 2010). Similar to the peak viscosity data, increasing the YP substitution significantly reduced the breakdown viscosity. This viscosity reduction is typical of composite flours, including ingredients rich in proteins and fibers (Mohammedet al., 2014, Thariseet al., 2014).

The setback viscosity is the viscosity of a paste cooled to 50 °C and held there for some time before the final viscosity is determined. The setback viscosity is correlated with the gelation process of the flour due to the recrystallization of the leached amylose from starch when the flour suspension is heated. A lower setback viscosity indicates less starch retrogradation. In the current study, the presence of YP increased the setback viscosity of the composite flour, although it was non-significant (p > 0.05). However, increasing the YP substitution led to the opposite trend, with a substantial decrease in setback viscosity at 15% YP substitution. Similarly, adding pumpkin seed cake to wheat flour has reduced the setback viscosity (Jukić et al., 2019).

The final viscosity represents the ability of composite flour to form a gel after cooking and cooling. The final viscosity data followed the peak viscosity trend and ranged between 2940 and 3607 cp. Increasing the YP substitution significantly reduced the final viscosity (p < 0.05) and gelling ability of the composite flour. The reduced final viscosity may be correlated with the dilution of starch, a lower degree of starch recrystallization, and lower gel hardness. Thus, a product with YP may have a softer and less brittle gel.

The composite flours showed significant reductions in the pasting temperature (p < 0.05) compared with the control wheat flour. The reduced pasting temperature suggests the extent of cooking at lower temperatures, which is favorable for minimizing energy consumption during food processing. Ashraf et al. (2020) reported a non-significant change in the pasting temperature for composite flours with lower substitution levels of pumpkin powder with other hydrocolloids.

### 4.3 Gel texture analysis

The hardness reduction suggests that the gels were more viscous. In a previous study, adding okra pod extract was found to reduce the firmness of wheat flour gels (Alamri 2014). Similarly, adding Cordia fruit polysaccharides was found to lower the hardness of wheat flour gels (Mahmood et al., 2018). Cohesiveness represents the internal bonds that hold the composite flour gels' structure together, indicating the degree of deformation due to an applied external compression. Adhesiveness indicates the degree of stickiness of the gels once a solid comes in contact. The presence of YP significantly improved the stickiness of the composite gels (p < 0.05).

However, the cohesiveness remained statistically the same for all gels regardless of the amount of YP substitution. Springiness represents the ability of a gel to retain its geometric shape after the external stress is removed. The least springy gel was with 10% YP. Gumminess is the product of hardness and cohesiveness. Thus, a reduction in the hardness would reduce the gumminess of the composite flour gels. Only the highest YP substitution level (15%) significantly reduced the gumminess (15.6) compared with the control gel. Mahmood et al. (2018) also reported that the gumminess of wheat flour was reduced by incorporating freeze-dried gum cordia. Thus, a softer wheat flour gel can be obtained by adding YP.

### 4.4 Micro-doughLab study

Costa et al. (2018) found that adding 30% pumpkin seed powder significantly reduced the water absorption of their composite dough. The dough development time is the water added to the first drop in the dough consistency. The development time varied from 1.4 to 9 min, and the composite doughs showed a significant increase in development time (p < 0.05) compared with the control dough. This may be due to the YP and wheat proteins competing for water and hindering each other, which delayed the hydration and dough development.

Also, Codină et al. (2019) reported that the dough development time increased with golden flaxseed flour in wheat flour. Conversely, the development time was reduced when gum Cordia and pumpkin seed powder were added to wheat flour (Costaet al., 2018, Mahmoodet al., 2018). Dough stability dictates the strength of the dough by retaining the maximum consistency over a long time under shear. In line with the development time, the stability of the composite doughs increased with YP substitution. The stability is an indirect indicator of the tolerance of the blended flour; hence, the composite doughs had a higher MTI than the control dough. Dough softening indicates the difference in the maximum consistency and consistency after 5 min. Composite flours showed more dough softening, which may be attributed to the dilution of gluten proteins by YP substitution. Codină et al. (2019) reported that the addition of brown flaxseed flour reduced the stability time of composite flours.

### 4.5 Bread quality

YP addition may have hindered the stretching of the thin-walled gluten cells during baking and reduced the volume. Solvita et al. (2015) also reported that the bread volume was reduced with pumpkin byproducts. The specific volume is the ratio between the volume and weight of the bread. An apparent reduction in the specific volume was observed. In line with the current study, El-Sohaimy et al. (2019) reported significant decreases in the volume and specific volume of flatbread when quinoa flour was added to the wheat flour. Moreover, Water activity is essential for the shelf stability of the bread. The composite flours resulted in bread with significantly higher water activity than the control bread. This may be attributed to the higher WHC of the combined flours. In addition to the volume and shape, the bread color defines its appearance and assists consumers with their purchase decisions.

### 4.6 Bread color

The quick and uniform formation of Maillard pigments in the baked layer may explain the relative darkness of the crust. In contrast, Costa et al. (2018) reported that their wheat-pumpkin seed bread was darker than the control. Similarly, the presence of YP reduced the redness of the crumb and yellowness as well. In the crumb case, the composite bread had increased lightness compared with the control bread. The reduced formation of pigments may be attributed to the lack of free proteins and the reduced availability of hydrolyzed saccharides.

In line with the crust data, the crumbs of the composite bread also showed reduced redness and yellowness, especially with increasing YP substitution. Conversely, the crust color was significantly darker for the composite bread than for the control bread (p < 0.05). It was reported that the addition of quinoa flour to the flatbread reduced the lightness, although the redness and yellowness were increased (El-Sohaimy et al., 2019). The composite bread with the maximum YP substitution (15%) had the highest crumb lightness, as well as the lowest redness and yellowness. The greater variations in the color parameters of both slices of bread may be attributed to the differences in processing.

### 4.7 Proximate composition

The most significant variation was observed with 5% and 10% YP substitutions, whereas increasing the substitution to 15% did not improve the prepared bread's proximate compositions. The increases in the fat and protein contents may be attributed to the higher fat and protein fractions of YP compared with those of wheat flour. In line with the current study, they enhanced protein, fat, and ash contents for their wheat-quinoa composite bread (El-Sohaimy et al., 2019).

### 4.8 Total phenols, antioxidants, and radical scavenging activity

The formation of Maillard reaction products in the control bread facilitates bioactivity in vitro that may augment the wheat phenolics and flavonoids (Gawlik-Dziki et al., 2009); this may be why the bioactivity was higher than in the composite bread. Conversely, Różyło et al., (2014) found that adding pumpkin flour increased the total phenolics and flavonoids in the bread. The 15% YP pan and pita bread had the highest DPPH antiradical activities of 6.95% and 6.40%, respectively. It was found that a strong positive correlation between the total phenolics and DPPH antioxidant activity (Kulczyński et al., 2020). However, the ABTS antiradical activity reached its maximum with 10% YP for both types of bread and significantly declined with 15% YP. Różyło et al. (2014) reported that their wheat-pumpkin composite bread had improved ABTS-based bioactivity compared with the control. The composite bread extracts' ability to reduce iron was estimated using FRAP assay. The FRAP activity significantly increased with YP substitution. However, the maximum increase was observed with 10% YP for both types of composite bread. The phenolics and carotene fractions in the YP may have imparted bioactivity to bread. Previously, they reported a positive correlation between the total phenolics and FRAP (Zheng and Wang, 2001). Recently, Kulczyński et al. (2020) reported a proportional increase in FRAP with DPPH from pumpkin pulp.

### 4.9 Sensory analysis

Incorporating YP did not compromise the crust color of both the pan and pita bread. However, significant modifications in the symmetry and uniformity were observed. The control pan bread had the highest break shred, whereas the lower volumes of the composite bread did not provide enough oven spring to break and shred the final loaf. The composite bread with 15% YP had the lowest break shred. The control and 5% YP bread for pita bread showed comparable break shreds. However, further increasing the YP substitution reduced the break shred. For the crumb, the composite pan and pita bread were significantly less liked by the panelists than the control bread. The higher WHC and relatively soggy crumb may have resulted in the less positive reception. The crumb grain develops by the uniform mixing and distribution of gas bubbles within the gluten network. The dilution of gluten in the composite flour retarded the OHC and uniformity of the grains. A poor loaf volume was observed with higher YP content, which may be an indirect indicator of an inconsistent and non-uniform grain structure for both types of bread.

Bread with a softer texture and flexibility is preferred; however, increasing the YP substitution reduced the volume, which indirectly increased the firmness of the composite bread. The pan and pita bread with 15% YP substitution had the least preferred textures. Ashraf et al. (2020) observed that panelists found the crumb grain more acceptable once xanthan and guar gum was added to their wheat-pumpkin composite bread. The bread flavor is affected by various acids, alcohols, and their derivatives, which are mainly produced during proofing or fermentation. The control bread was the most preferred for flavor, and increasing the YP substitution significantly reduced the positive reception to both composite slices of bread. Panelists found the 5% YP composite bread acceptable as the control bread. Codină et al. (2019) found that adding brown and golden flaxseed flour improved pan bread's appearance, color, flavor, and overall acceptability. Conversely, El-Sohaimy et al. (2019) found that supplementing flatbread with quinoa flour reduced the sensory attributes.

## 5 Conclusions

With the increasing popularity of ready-to-eat bakery products, enriching them with underutilized fruits and vegetables high in fiber and bioactive ingredients is an attractive approach to improving public health. This study blended YP with wheat flour to develop nutritionally rich pan and pita bread. The composite flours showed better WHC and softer gels. The composite flour pastes provided lower consistency and viscoelasticity. The presence of YP reduced the dough strength and stability, which lowered the pan bread volume. However, the nutritional quality of the composite bread was greatly improved with enhanced antioxidant activity. Additionally, a low YP substitution (5%) maintained the overall acceptability of the composite bread. Thus, underutilized pumpkin-based products can be suitable for enriching bakery products with enhanced nutritional quality and bioactivity.

## Declaration of Competing Interest

The authors declare that they have no known competing financial interests or personal relationships that could have appeared to influence the work reported in this paper.
